# Multimodal anatomy of the human forniceal commissure

**DOI:** 10.1038/s42003-022-03692-3

**Published:** 2022-07-25

**Authors:** Kevin Akeret, Stephanie J. Forkel, Raphael M. Buzzi, Flavio Vasella, Irmgard Amrein, Giovanni Colacicco, Carlo Serra, Niklaus Krayenbühl

**Affiliations:** 1 Department of Neurosurgery, Clinical Neuroscience Center, University Hospital Zurich and University of Zurich, Zurich, Switzerland; 2 Brain Connectivity and Behaviour Laboratory, Sorbonne Universities, Paris, France; 3 Donders Centre for Cognition, Radboud University, Thomas van Aquinostraat 4, 6525 GD Nijmegen, the Netherlands; 4 Centre for Neuroimaging Sciences, Department of Neuroimaging, Institute of Psychiatry, Psychology and Neuroscience, King’s College London, London, UK; 5 Departments of Neurosurgery, Technical University of Munich School of Medicine, Munich, Germany; 6 Division of Internal Medicine, University Hospital Zurich and University of Zurich, Zurich, Switzerland; 7 Institute of Anatomy, University of Zurich, Zurich, Switzerland; 8 Department of Health Sciences and Technology, ETH, Zurich, Switzerland; 9 Division of Pediatric Neurosurgery, University Children’s Hospital, Zurich, Switzerland

**Keywords:** Neuroscience, Neurophysiology

## Abstract

Ambiguity surrounds the existence and morphology of the human forniceal commissure. We combine advanced in-vivo tractography, multidirectional ex-vivo fiber dissection, and multiplanar histological analysis to characterize this structure’s anatomy. Across all 178 subjects, in-vivo fiber dissection based on the Human Connectome Project 7 T MRI data identifies no interhemispheric connections between the crura fornicis. Multidirectional ex-vivo fiber dissection under the operating microscope demonstrates the psalterium as a thin soft-tissue membrane spanning between the right and left crus fornicis, but exposes no commissural fibers. Multiplanar histological analysis with myelin and Bielchowsky silver staining, however, visualizes delicate cruciform fibers extending between the crura fornicis, enclosed by connective tissue, the psalterium. The human forniceal commissure is therefore much more delicate than previously described and presented in anatomical textbooks. This finding is consistent with the observed phylogenetic trend of a reduction of the forniceal commissure in non-human primates compared to non-primate eutherian mammals.

## Abstract (web summary)

Anatomical dissection and tractography elucidate the delicate nature of the human forniceal commissure, an interhemispheric white matter circuit.

## Introduction

Interhemispheric white matter circuits transverse the hemispheric midline and connect homotopic brain regions. Amongst these interhemispheric connections, the corpus callosum is the most prominent neopallial commissure in the human brain. Phylogenetically older commissures common to all vertebrates—the anterior commissure, ventral hippocampal, and forniceal (dorsal hippocampal) commissure—demonstrate a disproportionate reduction in primates^1–5^. While the anatomy and functions of the first two have been well described in the human brain^1,3–5^, the existence, morphology, and function of a forniceal commissure remains elusive.

The first anatomical descriptions of the forniceal commissure as a triangular subsplenial structure date back to macroscopic studies in monotremata and marsupialia in the late 19th century^6,7^. The forniceal commissure was first referred to as (dorsal) psalterium or David’s lyra, given its macroscopic similarity to the psalter, a zither-like instrument^6,7^. Subsequent terms included the dorsal hippocampal commissure^8^ – in contrast to the ventral hippocampal commissure – the fornix transversus^8^, forniceal commissure^9^, or the interammonic commissure^10^.

The first demonstration of the forniceal commissure in non-human primates (NHP) using silver impregnation methods emphasized the relative paucity of these fibers compared to non-primate eutherian mammals^11–20^. The striking reduction in its magnitude was confirmed during extensive investigations of the commissural connections of the hippocampal formation in macaques using axonal tracing^21–23^. These tracer studies showed that the fibers of the NHP forniceal commissure did not originate in the hippocampal formation proper but in parahippocampal areas: the periallocortical presubiculum and entorhinal cortex, as well as – albeit to a lesser extent – the isocortical and proisocortical areas of the posterior parahippocampal gyrus^21–23^.

In 1993, Gloor et al. published a comprehensive histological description of the hippocampal commissural system in humans^2^. While there was no significant remnant of the ventral hippocampal commissure, the dorsal hippocampal commissure (i.e., forniceal commissure) was described as a voluminous structure with a midline thickness corresponding to approximately one-fourth of the splenium^2^. This description of the forniceal commissure in humans distinctly exceeded that in NHP and thus contradicts the overall phylogenetic trend. It also contradicted electrophysiological studies, that implied the non-existence of a direct inter-hippocampal commissural connection in the human brain^24–26^. To the best of our knowledge, the study of Gloor et al. constitutes the only histological study of the human forniceal commissure to date^2^. More recent studies using ex vivo fiber dissection^27–30^ and in vivo tractography^30–37^ provided conflicting results regarding the existence and morphology of a forniceal commissure in the human brain.

In this multimodal anatomical study, we characterized the morphology of the human forniceal commissure by combining advanced in vivo tractography, multidirectional ex vivo fiber dissection, and multiplanar histological analysis.

## Results

### In vivo fiber dissection (tractography)

**Fig. 1:**
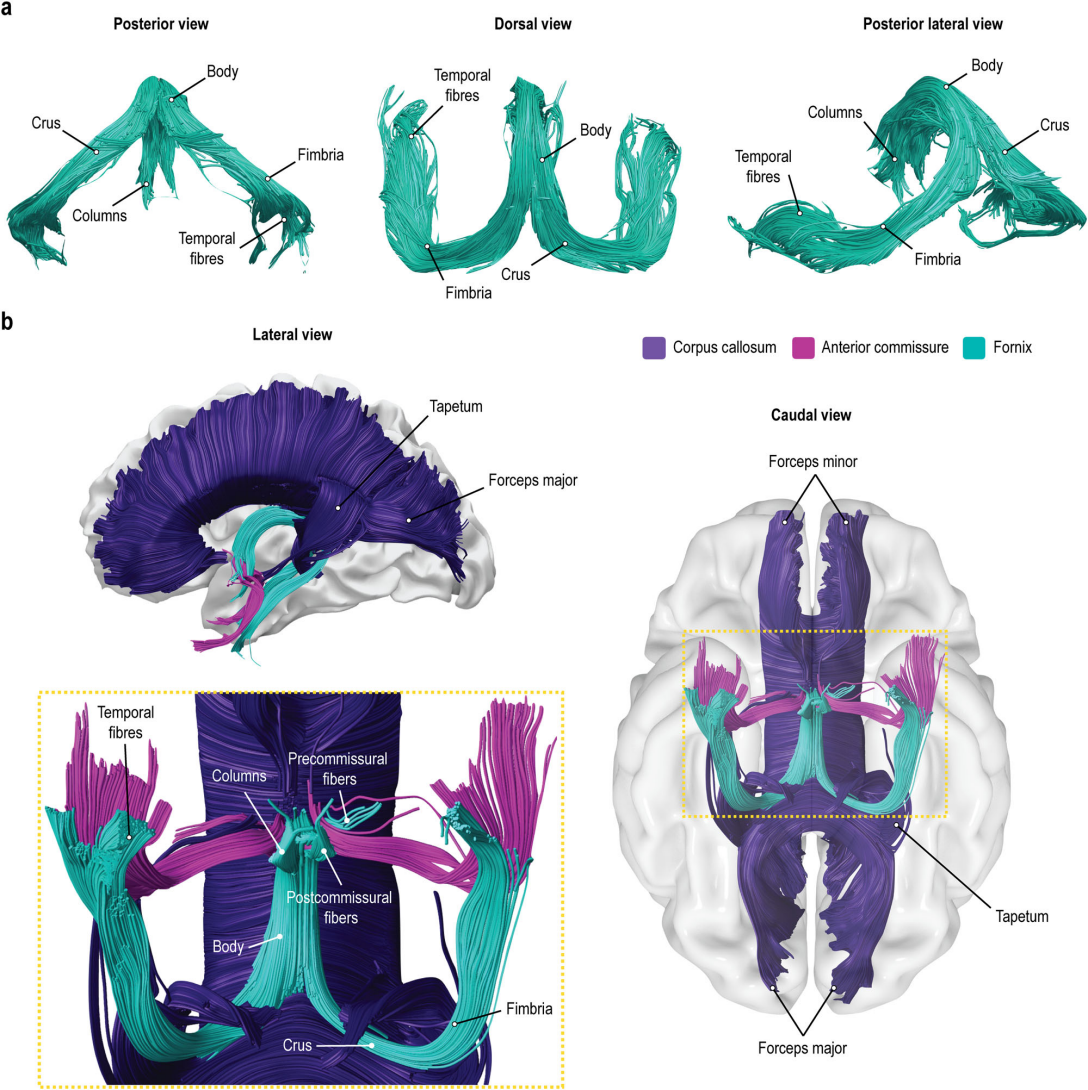
Commissural connections in relation to the fornix as identified with In vivo tractography dissections. Representative tractography streamline reconstruction of the fornix (**a**) and in relation to the corpus callosum and anterior commissure (**b**).

**Fig. 2:**
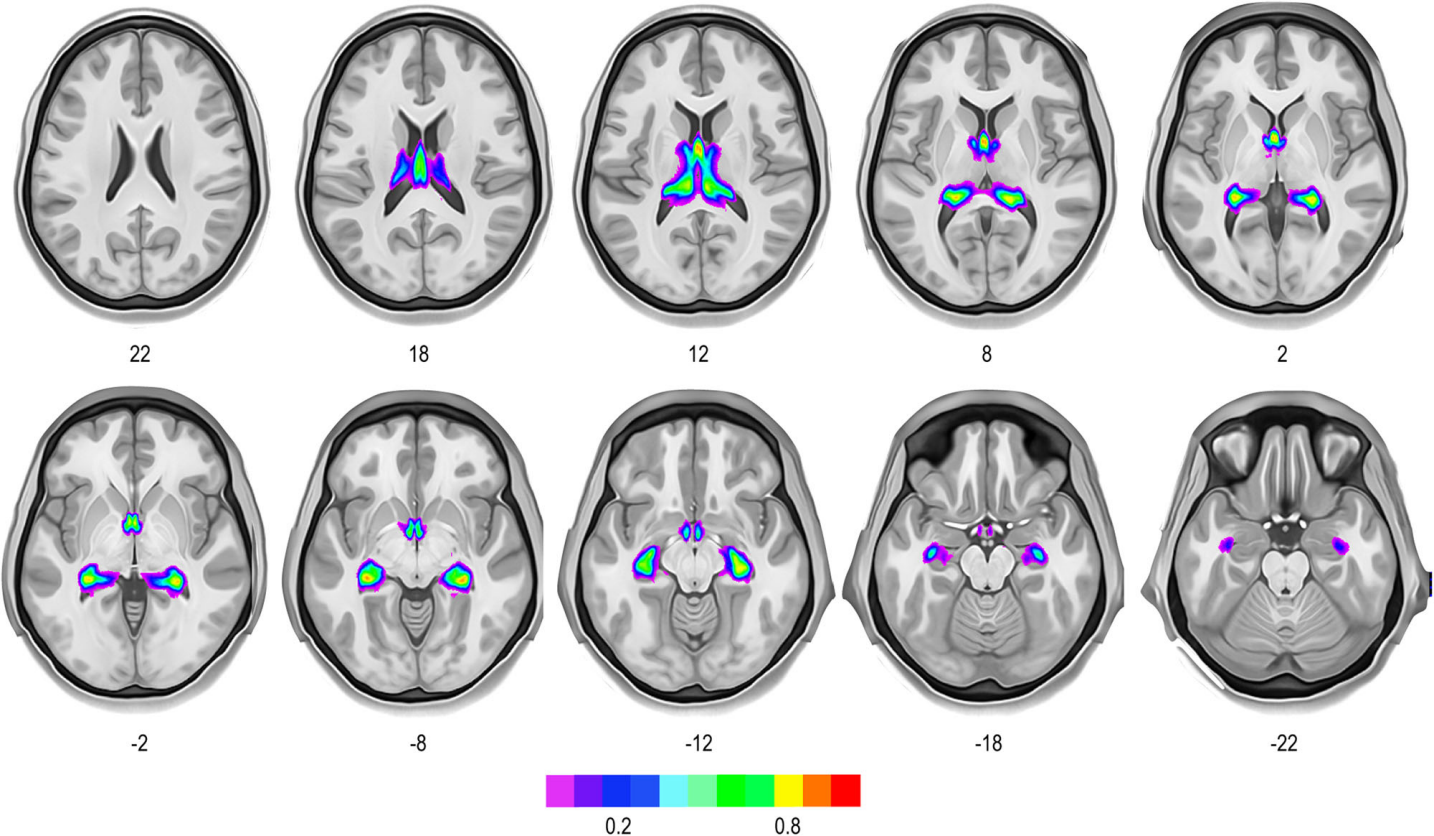
Percentage overlay map of the fornix for all 178 participants from the Human Connectome Project 7 T dataset. The full percentage overlay map of the fornices is available online (https://neurovault.org/collections/12108/).

We successfully identified and mapped the fornix in all 178 participants (individual dissections are available from https://neurovault.org/collections/12108). Figure 1 provides a representative tractography reconstruction of the fornix and its adjacent commissural white matter tracts. We reliably identified the anterior and posterior columns of the fornix enclosing the anterior commissure, the body of the fornix underneath the corpus callosum, and the split of fornix into the crura fornicis at the posterior end of the corpus callosum (splenium). The crura arched around the thalami and continued as fimbriae along the medial temporal lobe to terminate in the hippocampi. Commissural fibers between the crura fornicis were, however, not evident. Figure 2 shows the fornix percentage overlay map. The full map is provided online (https://neurovault.org/collections/12108/). There was no evidence of a forniceal commissure.

### Multidirectional ex vivo fiber dissection

The subsplenial region between the forniceal crura, where the forniceal commissure would be expected, was approached in a stepwise fashion. Fiber dissection was performed from ventral to dorsal, dorsal to ventral, and from caudal to rostral.

**Fig. 3:**
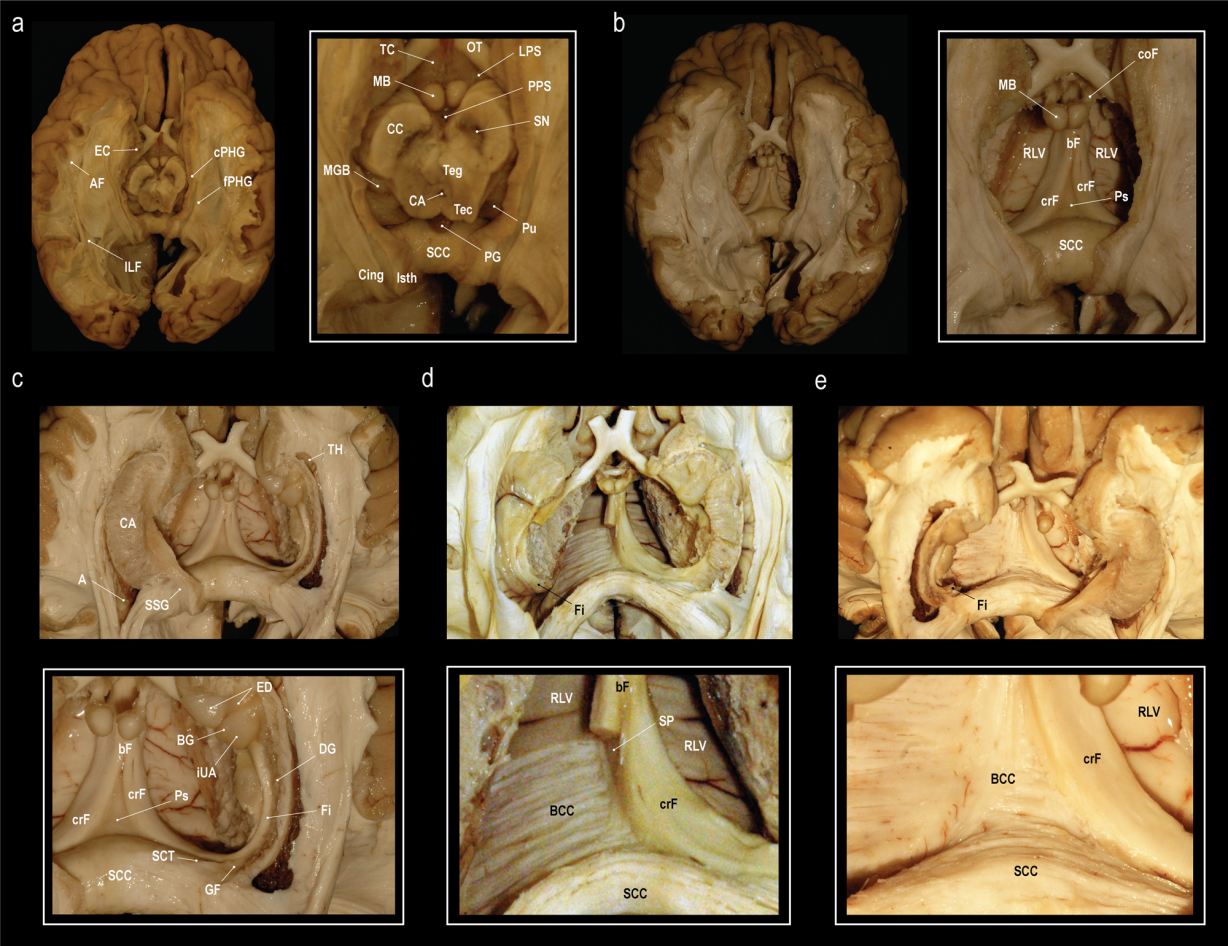
Fiber dissection from the ventral aspect. Stepwise fiber dissection of the subsplenial region from the ventral towards the dorsal surface of the cerebrum after brain preparation following a modified Klingler technique. **a** Exposure of the inferior longitudinal fasciculus (ILF) and the fibers of the parahippocampal gyrus (fPHG) after removal of the entorhinal cortex (EC), cortex of the parahippocampal (cPHG) and fusiform (lateral to the PHG, not shown) gyri and the underlying arcuate fibers (AF). CA cerebral aqueduct, CC crus cerebri, Cing cingulum, Isth isthmus, LPS lateral perforated substance, MB mammillary body, MGB medial geniculate body, OT optic tract, PG pineal gland, PPS posterior perforated substance, Pu pulvinar, SCC splenium of the corpus callosum, SN substantia nigra, TC tuber cinereum, Tec tectum mesencephali, Teg tegmentum mesencephali. **b** View on the roof of the lateral ventricles (RLV), crus (crF), body (bF) and column (coF) of the fornix, and the psalterium (Ps) after removal of the mesencephalon, thalamus, and parts of the hypothalamus and the caudate nucleus. **c** Exposure of the dentate gyrus (DG), fimbria (Fi), gyrus fasciolaris (GF), the subcallosal trigone (SCT), inferior surface of the uncal apex (iUA), band of Giacomini (BG), and the external digitations of the hippocampal head (ED) after removal of the fibers of the parahippocampal gyrus, inferior longitudinal fasciculus, part of the cornu ammonis (CA) and the ependyma of the floor of the atrium (A) and the temporal horn (TH). **d**, **e** Dissection of the fornix from the dorsal end of the fimbria (Fi) along the crus (crF) towards the body of the fornix (bF). Dissection of the body of the fornix (bF) reveals the septum pellucidum (SP). No commissural fibers between the fornicis were identified. Removal of the ependymal layer of the roof of the lateral ventricle (RLV) exposed the fibers of the body of the corpus callosum (BCC). *Dissections: NK*.

Dissection from ventral to dorsal (Fig. 3): The entorhinal cortex as well as the cortex from the parahippocampal and fusiform gyri was peeled away, and the arcuate fibers were removed to expose the fibers of the parahippocampus and the inferior longitudinal fasciculus (Fig. 3a). The mesencephalon, the thalamus, part of the hypothalamus and the caudate nucleus were removed to expose the columnae, body and crura of the fornices, and the roof of the lateral ventricles (Fig. 3b). To expose the fimbria, the fibers of the parahippocampal gyrus and the inferior longitudinal fasciculus were removed as well as part of the cornu ammonis and the ependyma of the floor of the atrium and temporal horn (Fig. 3c). The fornix was then dissected from the dorsal end of the fimbria along the crus towards the body (Fig. 3d). By the end of the dissection the body of the corpus callosum was exposed (Fig. 3e). We were able to identify the psalterium as a thin soft tissue membrane spanning between the right and left crus fornicis in all specimens. It was limited rostrally by the merging bodies of the fornix, rostro-dorsally by the septum pellucidum, dorso-caudally it attached to the splenium of the corpus callosum and ventrally it abutted the velum interpositum. We were, however, unable to identify any crossing fibers between the crura fornicis.

**Fig. 4:**
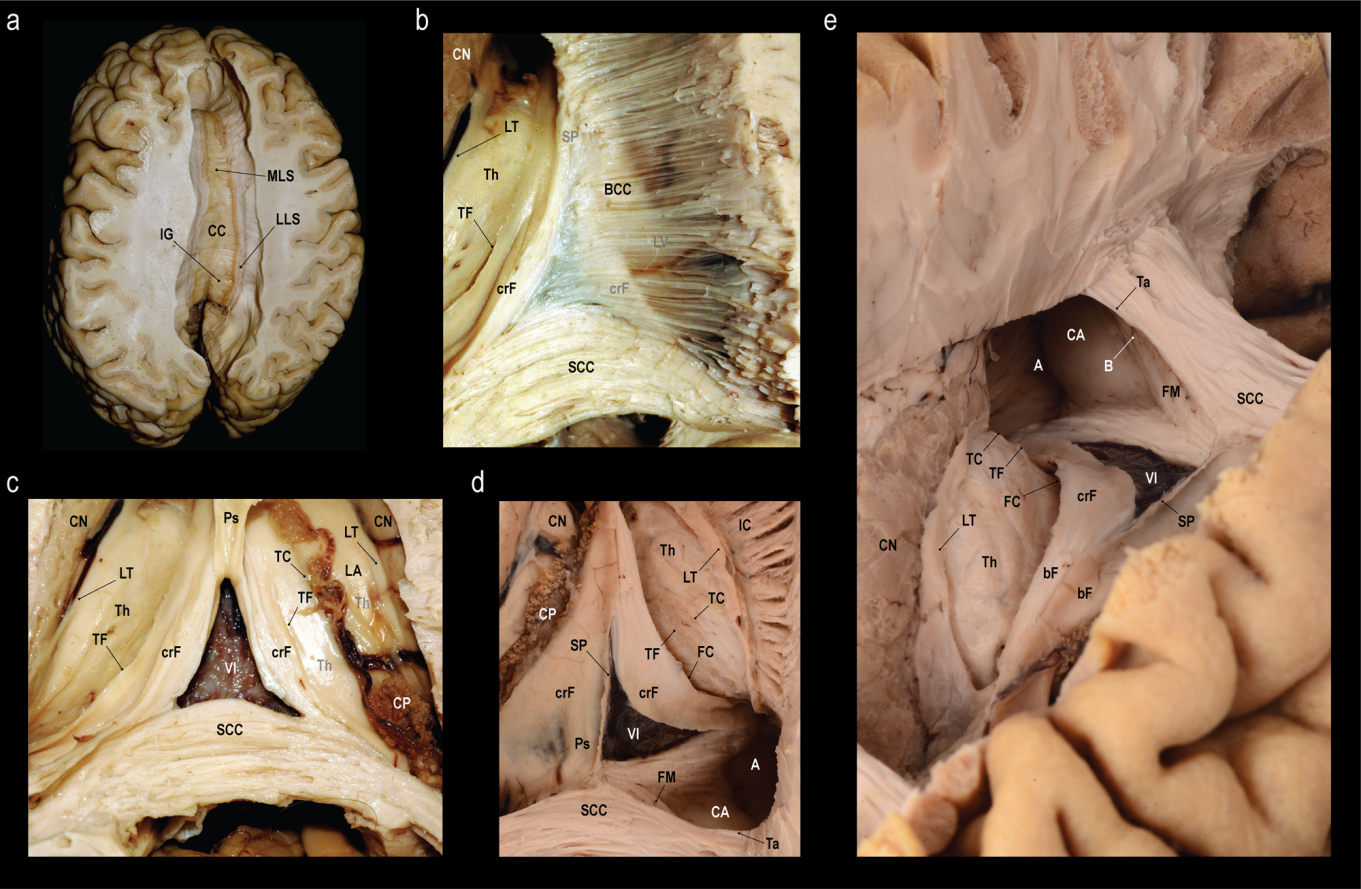
Fiber dissection from the dorsal aspect. **a** Exposure of the corpus callosum (CC) and the supracommissural hippocampus with its medial longitudinal stria (MLS), lateral longitudinal stria (LLS) and the indusium griseum (IG). **b** Removal of the fibers of the body of the corpus callosum (BCC) until the lateral ventricle (LV), the crus fornicis (CF), and the septum pellucidum (SP) were shining through (right). Further dissection of the remaining callosal fibers and removal of the choroid plexus and lamina affixa revealed the taenia fornicis (TF), thalamus (Th), lamina terminalis (LT) and the caudate nucleus (CN) (left). **c** Elevation of the psalterium (Ps) without identification of commissural fibers between the crura fornicis (CF) revealed the velum interpositum (VI). The choroid plexus (CP) is fixed to the crus fornicis via the taenia fornicis (TF) and to the lamina affixa (LA) through the taenia choroidea (TC). The thalamus (Th) is shining through the choroid plexus and the lamina affixa. **d** Different specimen, where the SP and Ps was left intact on the left side, but on the right the CP and LA were removed to expose the Th and the choroidal fissure (FC). Dissection of the right CN revealed the underlying fibers of the internal capsule (IC). Stripping of the ependyma in the atrium (A) exposed the fibers of the forceps major (FM). The calcar avis (CA) is formed by the underlying sulcus calcarinus. Starting from the SCC and running over the atrium are the fibers of the tapetum (Ta). **e** Anterior oblique view on the same specimen (before dissection of the CN). The fibers of the FM create the bulb of the posterior horn (B). *Dissections: NK (A-C) and CS (D-E)*.

Dissection from dorsal to ventral (Fig. 4): Both hemispheres were cut down to the level of the corpus callosum, exposing the supracommissural hippocampus. The callosal fibers were removed layer by layer until the ventricles, the septum pellucidum, and the crus fornicis were shining through. On one side the ventricle was opened to identify the caudate nucleus, the choroid plexus, and crus and body of the fornix. The removal of the remaining callosal fibers was performed and the underlying psalterium dissected without identification of crossing fibers between the fornices.

**Fig. 5:**
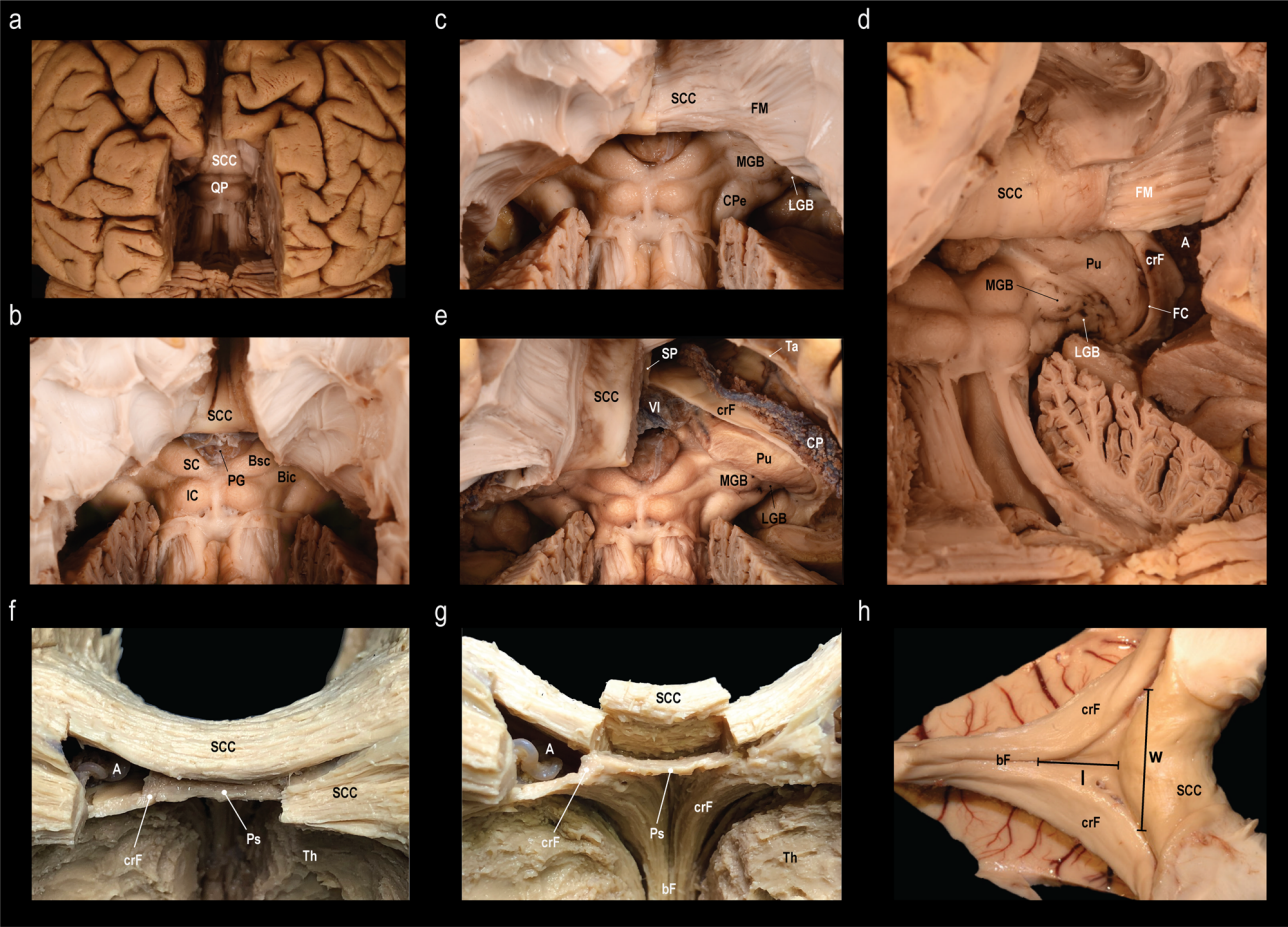
Fiber dissection from the caudal aspect and morphometry of the psalterium. **a** Exposure of the splenium of the corpus callosum (SCC) and the quadrigeminal plate (QP) after bilateral removal of the medial aspects of the occipital lobes and the cerebellar apex. **b** Closer view on the SCC, the pineal gland (PG), and the superior (SC) and inferior colliculi (IC) with the corresponding brachia (brachium of the superior colliculus, Bsc; brachium of the inferior colliculus; Bic) **c** Dissection of the subcortical fibers of the right isthmus revealed the fibers of the forceps major (FM) as caudo-lateral continuation of the SCC. In addition, the cerebral peduncle (CPe), medial geniculate body (MGB) and lateral geniculate body (LGB) are exposed. **d** Posterior oblique view on the crus fornicis (crF), the choroidal fissure (FC), and the pulvinar (Pu) after opening the atrium (A). **e** Midline incision and removal of the right half of the SCC exposes the septum pellucidum (SP), velum interpositum (VI), the tapetum (Ta) passing over the A, the crF, and the choroid plexus (CP). **f** Another specimen after fenestration of the SCC and partial removal of the thalamus (Th) to visualize the A, crF and psalterium (Ps). **g** Separation and elevation of the medial part of the SCC from the Ps. The unification of the crF into the body of the fornix (bF) form the anterior border of the Ps. **h** The length (l) of the Ps was measured from the unification of the crF to form the bF to the SCC. The width (w) of the Ps was defined as the distance between right and left attachment of the crF to the SCC. *Dissections: CS (A-E), KA (F-G), and NK (H)*.

Dissection from caudal to rostral (Fig. 5a–g): The hemispheres were reduced up to the splenium of the corpus callosum. The splenium was fenestrated to visualize the lateral ventricle, the crus fornicis and the psalterium. In the midline, the callosal fibers were separated and elevated from the psalterium. There were no fibers identified crossing from one crus fornicis to the other.

Based upon the measurements in nine human specimens (Table 1), the psalterium had a mean length of 1.4 cm (SD 0.40 cm) and a mean width of 2.4 cm (SD 0.51 cm) (Fig. 5).

**Table 1:** Morphometry of the psalterium.

Specimen	Length (cm)	Width (cm)
1	2.2	2.9
2	1.4	2.4
3	1.1	2.4
4	0.9	1.9
5	1.7	2.2
6	1.6	3.4
7	1.3	2.2
8	1.1	1.7
9	1.2	2.2

Length and width (compare Fig. 5) of the psalterium in nine human specimens.

In summary, the psalterium was visualized in all human brain specimens as a soft tissue membrane that spans between the two crus fornicis and is bounded rostrally by the union of the crus fornicis to form the body of the fornix, rostro-dorsally by the septum pellucidum, dorso-caudally by the splenium of the corpus callosum, and ventrally by the velum interpositum. It was not possible to identify commissural fibers between the two crura fornicis by further dissection of this membrane.

### Multiplanar histological analysis

**Fig. 6:**
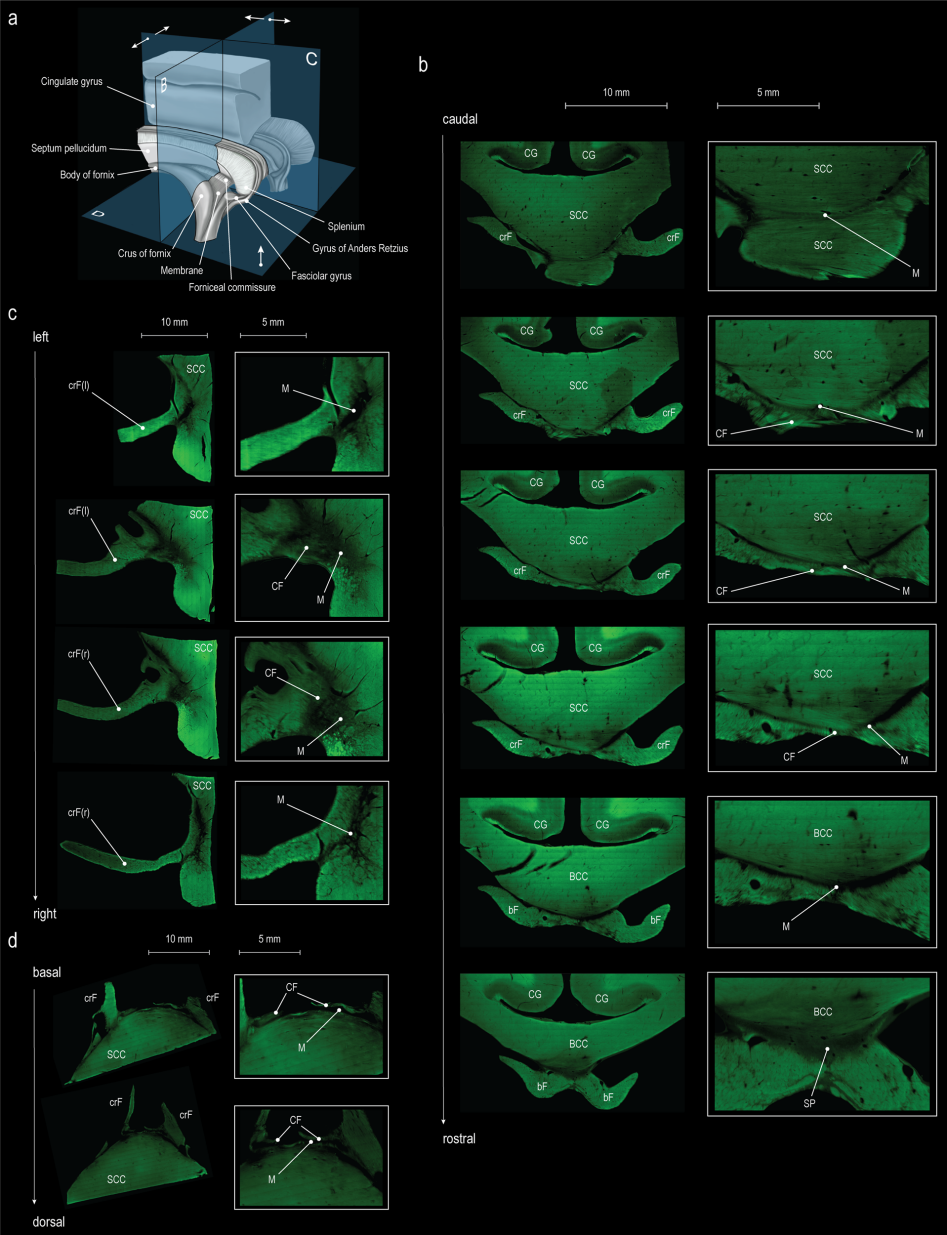
Multiplanar histology of the psalterium. **a** Schematic illustration of the histological section planes: coronal (B), sagittal (C) and axial (D). **b**–**d** Floating sections stained for myelin (1:900 dilution, FluoroMyelin Green, F34651, Thermo Fisher Scientific, MA, US) and counterstained with Hoechst 33342 (1:2000 dilution, H3570, Invitrogen, Carlsbad, CA): **b** Coronal sections from caudal to rostral. BCC body of the corpus callosum, bF body of the fornix, CF commissura fornicis, CG cingulate gyrus, crF crus fornicis, M connective tissue membrane, SCC splenium of the corpus callosum, SP septum pellucidum. **c** Sagittal sections from left to right. **d** Axial sections from basal to dorsal.

Representative examples of the myelin-stained histological appearance of coronal, sagittal and axial sections through the human splenium and caudal body of the corpus callosum are shown in Fig. 6. Supplementary Fig. 2 demonstrates a comparative study using the same tissue preparation and staining method in a sheep brain, in which the forniceal commissure is readily identifiable on macroscopic examination. Supplementary Fig. 3 shows representative coronally oriented serial sections through the human brain using the Bielschowsky silver staining technique.

While the forniceal (dorsal hippocampal) commissure was evident as a distinct und voluminous white matter tract between the two hippocampi in the sheep brain, in the human brain we identified only a delicate bundle of commissural fibers extending between the crura fornicis. While the splenial fibers showed a caudo-convex trajectory and were running in parallel bundles, the interforniceal fibers had a net-like configuration. In comparison to a previous histological description of the human forniceal commissure^2^, this commissural system was found to be very discreet and of small extent in the rostrocaudal axis. The caudal bulbous expansion of the splenium wrapped around the caudal contour of the forniceal commissure. Rostrally, the forniceal commissure formed an increasingly thinner plate fading out where the fornix detached itself from the undersurface of the corpus callosum and came together with the contralateral fornix. The commissural fibers were enclosed by connective tissue, which also formed the boundary to the splenium of the corpus callosum in the midline and the fibers of the forceps major laterally, corresponding to the macroscopically identified psalterium. Ventrally, the connective tissue was covered by pia mater, continuous with the pia mater of the fornices. Rostro-dorsally, the connective tissue layer merged into the septum pellucidum.

## Discussion

The multimodal anatomical approaches of this study indicate that the human forniceal commissure is much more delicate than previously described and presented in anatomical textbooks. This finding is consistent with the observed phylogenetic trend of a reduction of the forniceal commissure in NHP compared to non-primate eutherian mammals. This anatomical redimensionalization of the forniceal commissure permits a critical reinterpretation of previous studies and serves to tailor future investigations on the morphology, function, and pathophysiological role of this structure.

Virtual fiber dissection based on 7 Tesla diffusion-weighted imaging dataset of 178 participants did not identify interhemispheric connections between the crura fornicis. Previous tractography literature has been inconclusive about the visualization of a forniceal commissure: while some studies of the fornices did not describe commissural fibers^30,32–34^, others did describe a forniceal commissure^31,35,37,38^. Considering the spatial proximity between fibers of the splenium and the forniceal commissure, as histologically demonstrated in our study, there is a risk of misattributing splenial fibers as fibers of the forniceal commissure in tractography studies. The tractographic phenotype of the forniceal commissure in studies achieving putative detection^31,35,37,38^, resembles the visual appearance of rostrocaudal splenial fibers, but not that of histologically detected forniceal commissure fibers in our study. The absence of evidence in our study, however, might not be the evidence of absence. Previous reports indicated that fibers crossing the hemispheric midline have a different diameter^39^. In this case, the algorithm might not be able to trace commissural connections accurately. While the Human Connectome Project 7 Tesla data is a high-field high-resolution dataset offering high quality in vivo data (1 mm) for studying connectional anatomy in the living human brain, future studies might benefit from using submilimeter resolution datasets.

Our stepwise dissections of nine previously frozen, formalin-fixed human brains under the operating microscope showed a fine triangular soft tissue membrane between the forniceal crura, but no clear commissural fibers. The soft tissue membrane, which we identified in all specimens, was consistent with previous descriptions of the psalterium^29^. There is only a small number of fiber dissection studies specific to this anatomical region: no fibers suspicious of a forniceal commissure were identified by Shah et al. of 2012^27^, Destrieux et al. 2013^28^, and Güngör et al. 2017^30^. However, Tubbs et al. visualized the psalterium in twenty specimens, and in some cases dissected individual fine fiber-like bundles within the psalterium, which were designated as forniceal commissure^29^. We were not able to identify such fibers during the dissection under the operating microscope. While fiber dissection under the operating microscope after brain preparation following a modified Klingler technique appears to be suitable for identifying larger and parallel fiber tracts^40–43^, it might be less applicable to fine and cruciform fibers as is the case for the forniceal commissure. This may explain why we were unable to identify distinct commissural fiber tracts in the psalterium through ex vivo fiber dissection.

The microscopic anatomy of the forniceal commissure has been subject to extensive studies in NHP: the work of Amaral et al. on the commissural connections of the hippocampal commissure in macaques through the use of anterograde and retrograde labeling techniques revealed a number of differences in their organization compared to previous studies in rodents and lagomorphs^21^. First and foremost, there was an overall reduction in the size of the commissural projections. Second, it was surprising that the hippocampus proper received no commissural input. The most prominent portions of the commissural fibers originated in the presubiculum and terminated in the medial entorhinal area on the opposite side. The entorhinal cortex, in turn, was the origin of homotopic commissural projections with additional minor portions to the contralateral subiculum. Demeter et al. characterized the distinct hippocampal and parahippocampal origins of the fibers of fimbria, fornix, and the forniceal commissure in NHP^22,23^. From their presubicular, entorhinal, and parahippocampal origins, the fibers of the forniceal commissure pass through the alveus – not the fimbria – into the medial (alvear) fornix (contrasted by the lateral (fimbrial) fornix), continue to the undersurface of the crus fornicis, and thence cross along the undersurface of the splenium to the crus fornicis of the opposite side, where they follow the same path in a retrograde sequence. The morphological characteristics of the fibers comprising the forniceal commissure were studied in detail by Lamantia and Rakic in myelin-stained histological sections of primate brains^3^. In this work, the forniceal commissure was described to be composed of approximately 237,000 predominantly small and medium-sized myelinated axons, representing 0.4% of the telencephalic commissural axons. The fibers of the forniceal commissure could be differentiated morphologically from the fibers of the splenium by their smaller caliber and paler staining properties.

While early anatomical studies in humans contradicted the existence of a distinct forniceal commissure^17^, the work of Gloor et al. described a well-defined voluminous tract ventral to the splenium with fibers of smaller caliber and paler staining than those of the splenium^2^. Remarkable in the description by Gloor et al. was in particular the thickness of the tract, corresponding in its greatest extension to about one fourth of the splenium^2^. This surpasses the representation in NHP severalfold and contradicts the expected phylogenetic trend of a decrease in the volume of the forniceal commissures relative to the splenium^2,3,21–23^. In our multiplanar histological analysis, we reliably detected a subtle connective tissue membrane between the crura fornicis as the microscopic correlate of the psalterium. This membrane encapsulated fine cruciform fiber bundles, likely corresponding to the phylogenetic remnant of the forniceal commissure. In all six specimens, the forniceal commissure appeared much more discreet than described by Gloor et al.^2^, and as usually depicted in anatomical textbooks. The morphology of the structure identified as dorsal hippocampal commissure (i.e., forniceal commissure) in the work of Gloor et al.^2^ resembles the appearance of the rostrally turning ventral part of the splenium in our examinations.

There is considerable controversy in the literature not only regarding the morphological characteristics but also concerning the functional and pathophysiological relevance of the forniceal commissure^2,44^.

A detailed understanding of the anatomy of the human forniceal commissure, its relationship to the splenium of the corpus callosum, and its physiological radiomorphological appearance is important in the evaluation of commissural dysgenesis. Since the anterior commissure, the corpus callosum, and the forniceal commissure develop through the same commissural plate, malformations may often be combined and the popular term callosal agenesis falls short^29,45^. The forniceal commissure was described to be absent in complete callosal agenesis, which embryologically appears reasonable due to the shared commissural plate^29,45^. In partial agenesis, however, axonal rerouting through preexisting commissural substrates may result in a compensatory prominence of non-malformed commissures^46^. A compensatory prominent forniceal commissure (e.g., isolated callosal agenesis) may be confused with a preserved splenium of the corpus callosum (e.g., isolated splenium in holoprosencephaly)^29,45^. In patients with cavum septi pellucidi or cavum vergae, the psalterium, and hence also the forniceal commissure, was described to be absent, while the corpus callosum appears morphologically normal^29,47^. However, the multimodal anatomical characterization of the forniceal commissure in our study emphasizes that its discreet nature renders it almost impossible to draw definitive conclusions regarding its existence or prominence based on routine MRI or sonographic techniques. Further histological studies are needed to explore the relationship between ventricular development and the morphology of both psalterium and forniceal commissure.

Having a precise three-dimensional conception of a structure’s anatomy is the prerequisite for targeted and safe surgery. The forniceal commissure is of neurosurgical relevance, both as a potential target of ablative procedures and as a possible source of adverse effects in the event of unintended injury^29^. Transecting the psalterium has been advocated as an essential component of forebrain commissurotomy^48–51^. While certain authors attribute no or minor relevance to the forniceal commissure in mesial temporal lobe epilepsy^25,26,52–56^, others consider it as one of the most important pathways of contralateral seizure propagation and false lateralization of the ictal onset in extracranial EEG recordings^2,44,57,58^. Ictal involvement of the forniceal commissure has been associated with the phenomena of pure amnestic seizures and transient epileptic amnesia^2,44^. However, the specific contribution of transecting the psalterium to the anti-seizure effect of forebrain commissurotomy is still unclear. Since the functional relevance of the forniceal commissure remains obscure, it is unknown, which implications unintentional injury has. Memory deficits have been observed in patients after splenial callosotomies, yet it is unclear to what degree the forniceal commissure and the splenium, respectively, contributed to these deficits^59^. NHP were shown to suffer from impaired discrimination learning after lesioning the forniceal commissure^60^ and a recent study postulated a role of the forniceal commissure in human familiarity-based recognition memory^37^. Based on our macroanatomic fiber dissections and those of other groups^27–30,61^, however, it can be concluded that intraoperative visualization of the forniceal commissure, whether for preservation or ablation, is not a realistic prospect.

This study has several limitations that need to be considered. First, our sample size for the human ex vivo fiber dissection and histological analyses was relatively small with nine and eight specimens, respectively. While we believe that this sample size is sufficient for a general anatomical characterization, it did not allow any conclusions concerning the interindividual variability of this structure. This is further limited by the fact that the specimens were completely anonymized for legal reasons. Although neurological diseases were excluded prior to body donation, this prevented any conclusions regarding possible associations between demographic factors and the anatomy of the forniceal commissure. Second, our study lacks a morphometric analysis of the dorsoventral extent of the forniceal commissure and the psalterium. Reasons which rendered a macroscopic morphometric analysis inadmissible were the small dorsoventral extent of the structures bordering macroscopic measurement accuracy and the high variance of their extent along the rostrocaudal axis. And third, while we opted for a high resolution (HCP 7 T) in vivo analysis of the forniceal commissure, future studies might benefit from using submiliter resolution dataset to reinvestigate the fornix. The emergence of new datasets that combine postmortem and in vivo measurements might be a promising avenue toward mapping discreet anatomical structures^62,63^.

## Methods

This study was approved by the local ethical review board of the Canton of Zurich, Switzerland (KEK ZH 2016-00957).

### Nomenclature

As outlined above, the nomenclature of the structure examined in this study is complex for historical reasons. We used the descriptive terms *psalterium* and *forniceal commissure*. The psalterium was defined as a triangular subsplenial membrane extending between the crura fornicis. The term forniceal commissure was reserved for interforniceal fiber tracts. We chose to adhere to this nomenclature as the term dorsal hippocampal commissure can be considered misleading given the absence of fibers originating from the hippocampus proper in NHP and the absence of an identifiable ventral hippocampal commissure in humans.

### Imaging datasets

Structural connectome data were downloaded from http://www.bcblab.com/BCB/Opendata.html. This publicly available dataset was derived from the diffusion-weighted imaging dataset of 178 participants acquired at 7 Tesla by the Human Connectome Project Team (http://www.humanconnectome.org/)^64^. The Human Connectome Project 7 Tesla data is a high-field high-resolution dataset that is unique in its resolution to investigate connectional anatomy in the living human brain at 1 mm isotropic. Demographic cohort data are shown in Table 2. The scanning parameters have been described previously^64,65^. In brief, each diffusion-weighted imaging consisted of a total of 132 near-axial slices acquired with an acceleration factor of 3^66^, isotropic (1.05 mm3) resolution and coverage of the whole head with a TE of 71.2 ms and with a TR of 7000 ms. At each slice location, diffusion-weighted images were acquired with 65 uniformly distributed gradients in multiple Q-space shells^67^ and 6 images with no diffusion gradient applied. This acquisition was repeated four times with a b-value of 1000 and 2000 s/mm−2 in pairs with anterior-to-posterior and posterior-to-anterior phase-encoding directions. The default Human Connectome Project preprocessing pipeline (v3.19.0)^68^ was applied to the data^69^. The susceptibility-induced off-resonance field was estimated from pairs of images with diffusion gradient applied with distortions going in opposite directions^70^ and corrected for the whole diffusion-weighted dataset using TOPUP^71^. Subsequently, motion and geometrical distortion were corrected using the EDDY tool as implemented in FSL.

**Table 2:** Demographics of the human connectome project 7 tesla dataset.

Age	Sex		Total
	F	M	
22–25	1	20	21
26–30	50	35	85
31–35	56	14	70
36	2	0	2
Total	109	69	178

Age bins and sex distribution in the Human Connectome Project (downloaded from https://db.humanconnectome.org).

### In vivo fiber dissection

We performed whole-brain deterministic tractography in the native diffusion-weighted imaging space using StarTrack (https://www.mr-startrack.com). A damped Richardson-Lucy algorithm was applied for spherical deconvolutions^71,72^. A fixed fiber response corresponding to a shape factor of *α* = 1.5 × 10–3 mm2 s^−1^ was adopted, coupled with the geometric damping parameter of 8. Two hundred algorithm iterations were run. The absolute threshold was defined as three times the spherical fiber orientation distribution of a grey matter isotropic voxel and the relative threshold as 8% of the maximum amplitude of the fiber orientation distribution^73^. A modified Euler algorithm^74^ was used to perform the whole-brain streamline tractography, with an angle threshold of 35°, a step size of 0.5 mm and a minimum streamline length of 15 mm.

We co-registered the structural connectome data to the standard MNI 2 mm space using the following steps: first, whole-brain streamline tractography was converted into streamline density volumes where the intensities corresponded to the number of streamlines crossing each voxel. Second, a study-specific template of streamline density volumes was generated using the Greedy symmetric diffeomorphic normalization (GreedySyN) pipeline distributed with advanced normalization tools (ANTs)^74,75^. This provided an average template of the streamline density volumes for all subjects. The template was then co-registered with a standard 2 mm MNI152 template using flirt as implemented in FSL. This step produced a streamline density template in the MNI152 space. Third, individual streamline density volumes were registered to the streamline density template in the MNI152 space template and the same transformation was applied to the individual whole-brain streamline tractography using the trackmath tool distributed with the software package Tract Querier^76^ using ANTs GreedySyn. This step produced a whole-brain streamline tractography in the standard MNI152 space. Two examiners (KA, SJF) reviewed the alignment through visual inspection and ensured its match with the MNI152 template.

The fornix was dissected manually by two examiners (KA, SJF) in every individual dataset using Trackvis (www.trackvis.org). The placement of the regions of interest (ROIs) and regions of avoidance (ROAs) was guided by the high resolution T1-weighted image (Supplementary Fig. 1). We used an atlas^38^ based single spherical ROI around the body of the fornix, which was extended around the crus fornicis of each side, to avoid missing any commissural fibers. ROAs were defined for the corpus callosum and the anterior commissure, additional exclusion regions were placed anterior to the septum pellucidum and posterior to the splenium of the corpus callosum.

### Percentage overlay maps

The resulting reconstructions were binarized and converted to fiber density maps to generate percentage overlay maps using an in-house matlab script (Matlab R2021b). Results were visualized with FSL (https://fsl.fmrib.ox.ac.uk/fsl/fslwiki/FSLeyes).

### Cadaver specimens

17 human brains were obtained from healthy body donations to the Anatomical Institute of the University of Zurich, Switzerland. In Switzerland, body donations are fully anonymized beyond the confirmation of the absence of any neurological pathology, therefore no demographic data were available for the human specimens. Sheep brains for comparative macroscopic and histological post mortem studies were derived from autopsy specimens at the Animal Hospital Zurich from previously healthy female Swiss Alpine sheep (age 2–4 years).

### Multidirectional ex vivo fiber dissection

Nine brains were used for fiber dissection under the operating microscope. Brain preparation followed a modified version of the technique originally described by Joseph Klingler^77,78^: in brief, the fresh specimens were fixed in a 5% formalin solution for at least 2 months. After fixation, the leptomeninges were removed under the operating microscope. This was followed by refrigeration for 7 days at a temperature of −10 to −15 °C. After thawing, the brains were dissected^40,61,77,79^. The fiber dissection was performed from the superior and inferior aspect and from posterior in three specimens, respectively. Handmade soft wooden spatulas of various tip sizes, suction tips and microforceps were used to peel away the fibers. Specimen storage between different dissection steps was performed in 5% formalin solution at 5 °C.

### Morphometry of the psalterium

The length and the width of the psalterium were measured in the nine brain specimens used for fiber dissection under the operating microscope using a surgical caliper. The length (l) of the psalterium was measured from the unification of the crura fornicis to form the body of the fornix to the splenium of the corpus callosum. The width was defined as the distance between the right and left attachment of the crura fornicis to the splenium of the corpus callosum. A morphometric analysis of the thickness (dorsoventral extent) of the forniceal commissure or the psalterium was not attempted, given the small dorsoventral extent of the structures bordering macroscopic measurement accuracy and the high variance of their extent along the rostrocaudal axis.

### Multiplanar histological analysis

Eight formalin-fixed human brains and two sheep brains were used for histological analysis. Six human brains were cut through the splenium and caudal body of the corpus callosum into serial floating sections of 120 µm thickness with a mounting interval of 360 µm using a Vibratome (VT1000 S, Leica, Switzerland) oriented in the coronal (2x), sagittal (2x), and axial (2x) plane (Fig. 6a). Given the already macroscopically evident forniceal commissure, the sections in the sheep brain were oriented in the coronal plane only (2x). The floating sections were stained for myelin (1:900 dilution, FluoroMyelin Green, F34651, Thermo Fisher Scientific, MA, US) and counterstained with Hoechst 33342 (1:2000 dilution, H3570, Invitrogen, Carlsbad, CA, US) for 40 min at room temperature. After three washing steps for 20 min with phosphate buffered saline, the sections were mounted using ProLong Gold (Invitrogen, Carlsbad, CA, US). Two brains were used for coronally oriented paraffin sections of 8 µm thickness. Bielchowsky silver staining (Bielschowsky Silver Stain Kit, ab245877, abcam, Cambridge, UK) was performed for representative sections according to the manufacturer’s protocol. In brief, the sections were deparaffinized, hydrated in distilled water, incubated for 15 min at 40 °C in a silver nitrate solution (20%), washed with distilled water in four changes, incubated for 10 min at 40 °C in ammoniacal silver solution, then placed into the developer solution for some seconds until tissue sections took on a yellow/brown hue, followed by a placement in ammonia water for 30 s, four washing steps with distilled water, incubation for 2 min in sodium thiosulfate solution (5%), dehydration, and mounting with Permount medium (SP15, Thermo Fisher Scientific, MA, US). Whole-slide scans of the histological sections were produced by merging single images obtained with a Zeiss Observer Z1 microscope coupled to a motorized stage (Carl Zeiss AG, Feldbach, Switzerland) at 10x magnification.

### Reporting summary

Further information on research design is available in the Nature Research Reporting Summary linked to this article.

## Supplementary information

Supplementary Material

Reporting Summary

## Data Availability

The tractography data (Package X) is freely available as a preprocessed dataset from http://www.bcblab.com/BCB/Opendata.html. Individual dissections and the percentage overlay map of the fornices are available from https://neurovault.org/collections/12108/. A 3D model of the fornix is available for digital exploration and 3D printing at https://www.thingiverse.com/stephforkel/designs.
