# Supplementary material for: Multimodal anatomy of the human forniceal commissure
Source: Commun Biol. 2022 Jul 25;5:742. doi: 10.1038/s42003-022-03692-3 (PMC9314404; doi:10.1038/s42003-022-03692-3)
Supplement: Supplementary file 1 — Supplementary Material [file 42003_2022_3692_MOESM1_ESM.pdf]

Supplementary Information

Multimodal anatomy of the human forniceal commissure

Kevin Akeret[1], Stephanie J. Forkel[2,3,4,5], Raphael M. Buzzi[6], Flavio Vasella[1], Irmgard Amrein[7,8], Giovanni Colacicco[7], Carlo Serra[1], Niklaus Krayenbühl[1,9]

1 Department of Neurosurgery, Clinical Neuroscience Center, University Hospital Zurich and University of Zurich, Zurich, Switzerland.
2 Brain Connectivity and Behaviour Laboratory, Sorbonne Universities, Paris, France.
3 Donders Centre for Cognition, Radboud University, Thomas van Aquinostraat 4, 6525 GD Nijmegen, the Netherlands
4 Centre for Neuroimaging Sciences, Department of Neuroimaging, Institute of Psychiatry, Psychology and Neuroscience, King's College London, London, UK
5 Departments of Neurosurgery, Technical University of Munich School of Medicine, Munich, Germany.
6 Division of Internal Medicine, University Hospital Zurich and University of Zurich, Zurich, Switzerland.
7 Institute of Anatomy, University of Zurich, Zurich, Switzerland.
8 Department of Health Sciences and Technology, ETH, Zurich, Switzerland.
9 Division of Pediatric Neurosurgery, University Children's Hospital, Zurich, Switzerland.

Corresponding author:

Niklaus Krayenbühl
Division of Pediatric Neurosurgery and Department of Neurosurgery
University Children's Hospital and University Hospital of Zurich
Steinwiesstrasse 75
CH-8032 Zurich, Switzerland
Email: niklaus.krayenbuehl@kispi.uzh.ch

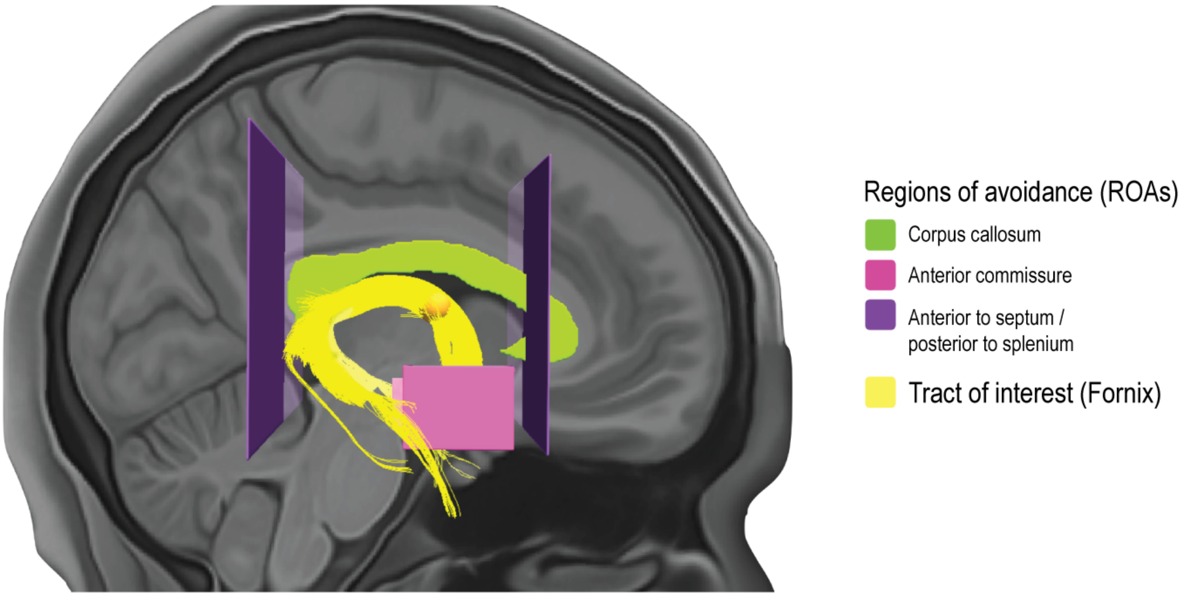

Supplemental Figure 1. Regions of interest (ROIs) and avoidance (ROAs).

Sagittal T1-weighted MR image exemplifying the ROIs and RAOs.

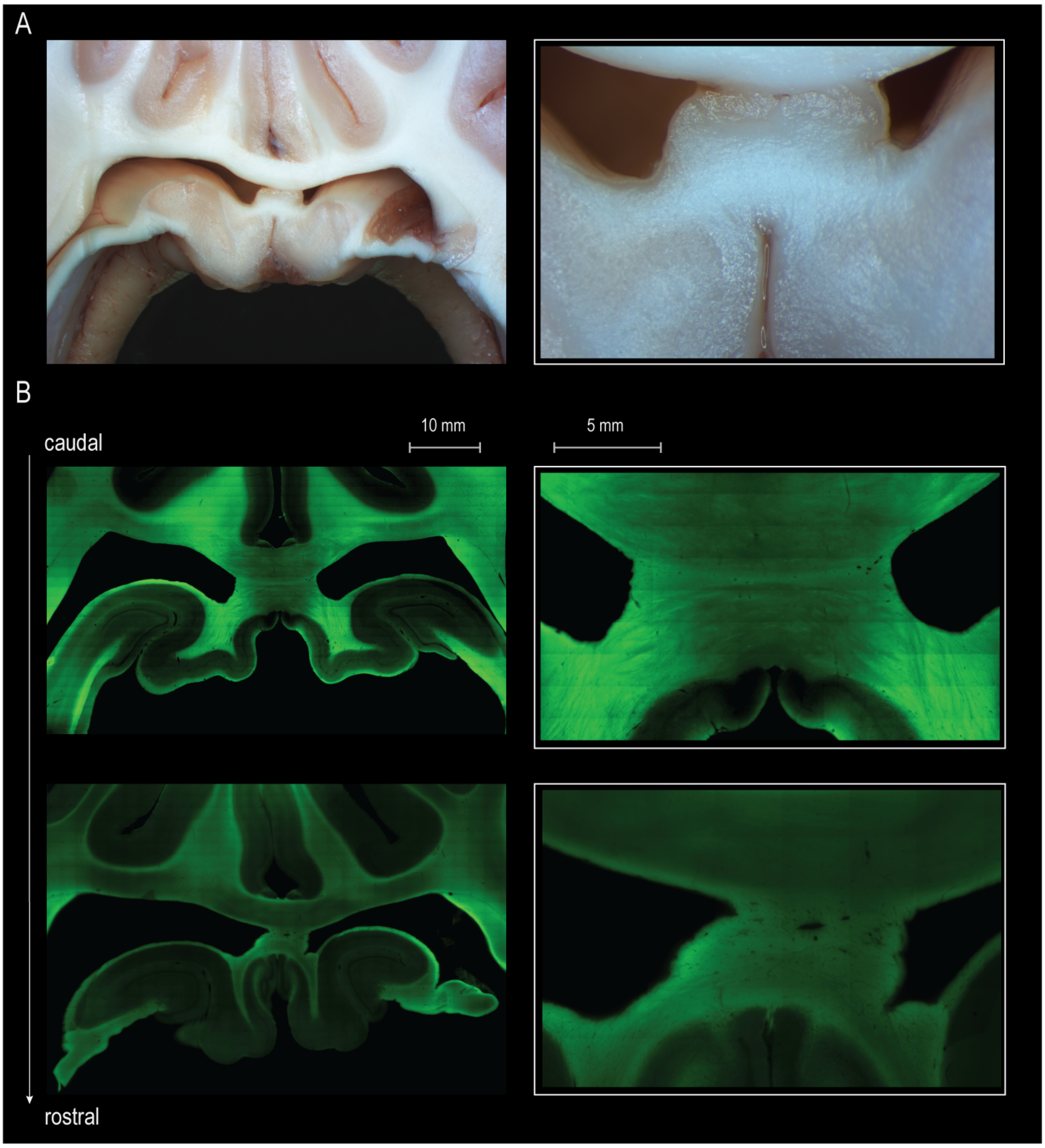

Supplemental Figure 2. Forniceal commissure in a sheep:

A. Macroscopic appearance of the forniceal commissure in a sheep. **B.** Coronal floating sections from caudal to rostral stained for myelin (1:900 dilution, FluoroMyelin Green, F34651, ThermoFisher, MA, US) and counterstained with Hoechst 33342 (1:2000 dilution, H3570, Invitrogen, Carlsbad, CA).

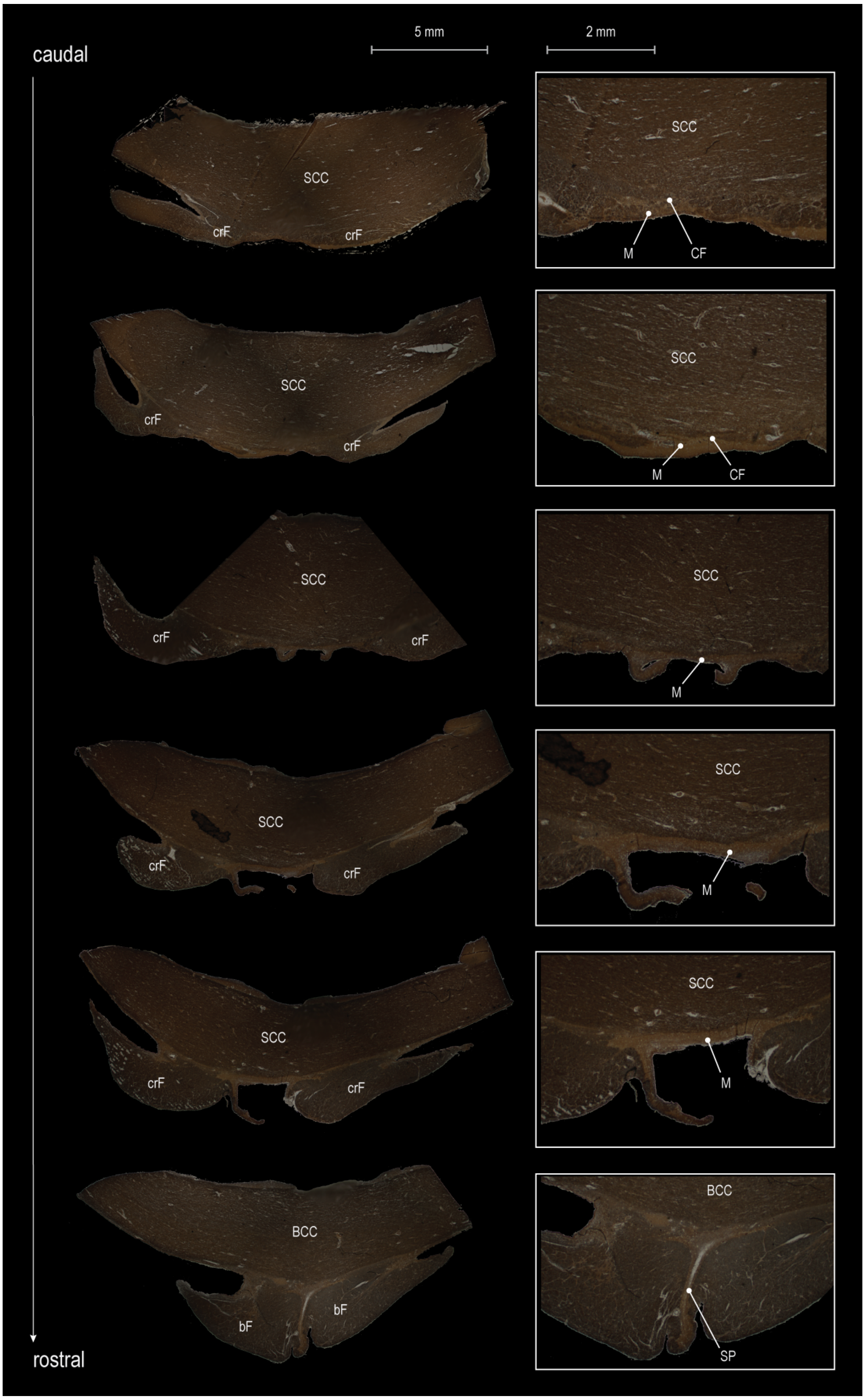

Supplemental Figure 3. Bielschowsky silver stain in human specimens:

Coronal paraffin sections from caudal to rostral with Bielschowsky silver staining (Bielschowsky Silver Stain Kit, ab245877, abcam, Cambridge, UK). BCC, body of the corpus callosum; bF, body of the fornix; CF, commissura fornicis; crF, crus fornicis; M, connective tissue membrane; SCC, splenium of the corpus callosum; SP, septum pellucidum.
